# The multiple mediating effects of vision-specific factors and depression on the association between visual impairment severity and fatigue: a path analysis study

**DOI:** 10.1186/s12888-024-06014-5

**Published:** 2024-08-21

**Authors:** Wouter Schakel, Christina Bode, Peter M. van de Ven, Hilde P. A. van der Aa, Carel T. J. Hulshof, Gerardus H. M. B. van Rens, Ruth M. A. van Nispen

**Affiliations:** 1 Amsterdam UMC Location Vrije Universiteit Amsterdam, Ophthalmology, PO Box 7057, De Boelelaan 1117, Amsterdam, 1007 MB the Netherlands; 2 Amsterdam Public Health, Quality of Care, Mental Health, Aging and Later Life, Amsterdam, the Netherlands; 3 University of Twente, Psychology, Health and Technology, Enschede, the Netherlands; 4 UMC Utrecht, Data Science and Biostatistics, Utrecht, the Netherlands; 5 Robert Coppes Foundation, Expertise Innovation Knowledge, Vught, the Netherlands; 6 Lighthouse Guild, New York City, NY USA; 7 Amsterdam UMC Location Academic Medical Center, Coronel Institute of Occupational Health, Amsterdam, the Netherlands

**Keywords:** Visual impairment, Fatigue, Depression, Vision-specific quality of life, Low vision, Structural equation modeling

## Abstract

**Background:**

Severe fatigue is a common symptom for people with visual impairment, with a detrimental effect on emotional functioning, cognition, work capacity and activities of daily living. A previous study found that depression was one of the most important determinants of fatigue, but less is known about disease-specific factors in this patient population. This study aimed to explore the association between visual impairment severity and fatigue in adults with low vision, both directly and indirectly, with vision-specific factors and depression as potential mediators.

**Methods:**

Cross-sectional data were collected from 220 Dutch low vision service patients by telephone interviews. Fatigue was defined as a latent variable by severity and impact on daily life. Potential mediators included vision-related symptoms, adaptation to vision loss and depression. Hypothesized structural equation models were constructed in Mplus to test (in)direct effects of visual impairment severity (mild/moderate, severe, blindness) on fatigue through above mentioned variables.

**Results:**

The final model explained 60% of fatigue variance and revealed a significant total effect of visual impairment severity on fatigue. Patients with severe visual impairment (reference group) had significantly higher fatigue symptoms compared to those with mild/moderate visual impairment (β = -0.50, 95% bias-corrected confidence interval [BC CI] [-0.86, -0.16]) and those with blindness (β = -0.44, 95% BC CI [-0.80, -0.07]). Eye strain & light disturbance, depression and vision-related mobility mediated the fatigue difference between the severe and mild/moderate visual impairment categories. The fatigue difference between the severe visual impairment and blindness categories was solely explained by eye strain & light disturbance. Moreover, depressive symptoms (β = 0.65, *p* < 0.001) and eye strain & light disturbance (β = 0.19, *p* = 0.023) were directly associated with fatigue independent of visual impairment severity.

**Conclusions:**

Our findings indicate an inverted-U shaped relationship between visual impairment severity and fatigue in patients with low vision. The complexity of this relationship is likely explained by the consequences of visual impairment, in particular by strained eyes and depressive mood, rather than by severity of the disability itself.

**Supplementary Information:**

The online version contains supplementary material available at 10.1186/s12888-024-06014-5.

## Background

Fatigue in visual impairment is described by patients as a daily, uncontrollable sensation with feelings of mental and physical exhaustion [1]. Evidence from recent meta-analyses with 14 studies indicates that fatigue severity levels and the odds of fatigue are higher in adults with visual impairment compared to normally sighted controls, with small to medium effect sizes reported [2]. Other results showed that symptoms of severe fatigue were present in 57% of adults with visual impairment which is at least twice as high compared to the general Dutch population [3]. We found that consequences of severe fatigue not only affect patients’ lives, but also pose an economic burden for society at large through loss of work participation [3]. To our knowledge, in-depth analyses investigating underlying mechanisms that may explain the association between visual impairment and fatigue are lacking.

Qualitative insights indicate that multiple factors may play a role in the onset and course of fatigue in patients with visual impairment. High cognitive load due to necessary adjustments for functioning in daily life, intensity of light, negative cognitions with regard to vision loss and the effort necessary for visual perception were among the most important causes of fatigue mentioned by visually impaired adults with severe fatigue symptoms [1]. In more recent work, we developed multidimensional path models using structural equation modelling (SEM) to explore psychological and health-related factors as determinants of fatigue in adults with visual impairment and adults with normal sight. The results indicate that fatigue in visual impairment is directly associated with depressive symptoms, and to a lesser extent with perceived health, poor somatic comorbidity and flexible goal adjustment coping tendencies [4]. Depressive symptoms and perceived health were identified as mediators in the relationship between sleep disorders, self-efficacy and fatigue. Due to the nature of the comparison with healthy adults, these path models did not include vision-specific factors that have been mentioned as important causes of fatigue by persons with visual impairment [1]. Knowledge of disease-specific fatigue factors may aid healthcare professionals to develop and tailor interventions to the needs of the target population. Therefore, the first objective of the present study was to explore vision-specific factors (e.g. eye strain, light disturbance) as determinants of fatigue severity and the impact of fatigue on daily life within a sample of adults with visual impairment. The influence of depression was also examined in the present study because it is a common symptom in patients with visual impairment [5] that also shares a strong relationship with fatigue [6, 7] and vision-specific factors [8–10].

Furthermore, there are some indications that poor visual acuity in adults with visual impairment may be linked to higher fatigue symptomatology. Two studies with objective measures of visual acuity found that older adults with moderate to severe visual impairment had higher fatigue levels than older adults with mild visual impairment [11, 12]. Some other population-based studies with self-reported measures have also reported higher levels of fatigue symptomatology for older adults who rated their vision to be poorer [13, 14]. However, this evidence is based on a small number of studies and conflicting results have been reported by Williams et al. [15], who found that persons with legal blindness in both eyes experienced less fatigue relative to persons with moderate visual impairment in the better eye. These findings suggest that vision loss may not directly influence fatigue, but may play a role through an interplay with other related factors. In the present study we therefore explored the association between visual impairment severity and fatigue and examined whether this link may be mediated by other vision-specific factors.

## Methods

### Design and participants

Data for the present study were collected as part of a larger cross-sectional survey on fatigue among patients with visual impairment. Two related studies on this project have been previously published, including an economic evaluation of the burden of visual impairment and comorbid fatigue [3], and a path analysis of generic factors that determine fatigue in adults with and without visual impairment [3]. A random sample of 1281 patients with visual impairment who were registered and received care at two low vision services in the Netherlands (Royal Dutch Visio and Bartiméus) at the time of the study (2015–2016) were invited by letter to participate. Patients with at least mild visual impairment according to the World Health Organization (WHO) criteria (defined as presenting visual acuity (VA) worse than 20/40 (6/12, 0.50) and/or concentric visual field impairment worse than < 45° in the better-seeing eye), sufficient mastery of the Dutch language and who were 18 years of age or older were eligible. Exclusion criteria were severe cognitive impairment, as defined by 3 or more errors on the 6-item version of the Mini Mental State Examination (MMSE) [16], and a diagnosis or receiving treatment in the last year for the following chronic conditions of which fatigue is a common symptom: cancer, multiple sclerosis, chronic fatigue syndrome and psychiatric disorders. Patients who agreed to participate completed a battery of validated questionnaires and gave information about socio-demographic and clinical characteristics through a structured telephone interview (performed by two experienced researchers with MSc and BSc in psychology).

### Measures and data preparation

Socio-demographic characteristics collected include age, gender, living situation (living alone vs. living together with a partner or family), education and employment status. Somatic comorbidity was defined as having no comorbidity or being treated for one or more of seven comorbid chronic conditions: asthma or chronic obstructive pulmonary disease; osteoarthritis and rheumatoid arthritis; peripheral arterial disease; diabetes mellitus; cardiac disease; cerebrovascular accident or stroke; cancer; and other chronic somatic or psychiatric conditions.

Data on visual acuity, visual fields, ophthalmic diagnoses and other descriptions of vision loss and/or visual field impairments were obtained from patient medical records at low vision services, and were used to supplement missing values. In accordance with the WHO criteria, four categories of visual impairment were defined based on the better-seeing eye. Mild visual impairment referred to presenting VA worse than 20/40 (6/12, 0.50) and equal or better than 20/60 (6/18, 0.33), or concentric visual field impairment < 45° and ≥ 0.30. Moderate visual impairment referred to presenting VA worse than 20/60 (6/18, 0.33) and equal or better than 20/200 (6/60, 0.10), or concentric visual field impairment < 30° and ≥ 20°, or loss of upper visual field/hemianopia. Severe visual impairment referred to presenting VA worse than 20/200 (6/60, 0.10) and equal or better than 20/400 (6/120, 0.05), or concentric visual field impairment < 20°. Blindness (societal and total blindness) referred to presenting VA worse than 20/400 (6/120, 0.05) or concentric visual field impairment < 10°. Because mild visual impairment was only present in 9 patients, moderate and mild visual impairment were combined into one category for SEM analyses.

The Fatigue Assessment Scale (FAS) [17], Modified Fatigue Impact Scale (MFIS) [18], Patient Health Questionnaire (PHQ-9) [19] and Adaptation to Vision Loss questionnaire (AVL-9) [20, 21] were analyzed with item response theory (IRT) models (i.e. the graded response model) to ensure these measures had satisfactory psychometric properties using R studio version 1.1.456, and R version 3.5.1. Questionnaires were adjusted based on their performance in these statistical models, followed by calculation of respondents’ thetas, which reflect an interval score of the underlying trait. This procedure has already been described in great detail in our previous path analysis study [4]. IRT outcomes with fit indices, questionnaire adjustments and theta parameters are available in Supplement 1. All other questionnaires used for independent, potential mediating and latent variables in SEM analyses are shown in Table 1.

**Table 1:** Questionnaires, characteristics and outcomes for variables used in SEM analyses (*n* = 220)

Variable	Measure	Characteristics			Outcomes		
		Items	Range	Response options	Type	M	SD
Fatigue severity	Fatigue Assessment Scale (FAS)	10	10–50	Never (1) – always (5)	Theta	0.34	0.87
Fatigue impact on daily life	Modified Fatigue Impact Scale (MFS)	21	0–84	Never (0) – almost always (4)	Theta	0.47	1.04
Depressive symptoms	Patient Health Questionnaire (PHQ-9)	9	0–27	Not at all (0) – nearly every day (3)	Theta	0.44	0.78
Adaptation to vision loss	Adaptation to Age-Related Vision Loss (AVL-9)	9	0-27^a^	Strongly agree (0) – strongly disagree (3)	Theta	-0.01	0.85
Mobility difficulties due to visual disability	Mobility subscale of Low Vision Quality Of Life questionnaire (Dutch LVQOL 21-items adapted version)	5	0-100^b^	Not able because of my vision (0) – no problem (5)	Sum score	49.9	19.0
Eye strain and light disturbance	Latent variable comprised of three LVQOL items^c^ and one item^d^ of the Dutch ICF Activity Inventory (D-AI) feeling fit subscale	4		LVQOL: Not able because of my vision (0) – no problem (5)	Theta	-0.01	1.03
				D-AI: Not difficult (0) – impossible (4)			

^a^A higher score indicates better adaptation

^b^A higher score indicates less mobility difficulties

^c^“*How much of a problem do you have: getting the right amount of light to be able to see*”, “*How much of a problem do you get: with your eyes getting tired (e.g. only being able to do a task for a short period of time)*”, “*How much of a problem do you get: with glare (e.g. dazzled by car lights or the sun)*”

^d^“*How difficult is it for you to: perform your daily activities without suffering from discomfort in the eyes (e.g. eye strain)”*

In accordance with the model of our previous path analysis study [4], a latent fatigue variable was defined by two indicators: fatigue severity (FAS) and fatigue impact (MFIS). As a measure for eye strain & light disturbance, a latent variable was created from three Low Vision Quality of Life questionnaire (LVQOL) [22, 23] items and one item of the Dutch ICF Activity Inventory (D-AI) feeling fit subscale [24], because, to the best of our knowledge, no specific questionnaires regarding these concepts were available. LVQOL item “how much of a problem do you have: getting the right amount of light to be able to see” was removed due to poor factor loadings and strong collinearity, resulting in a three factor latent variable that was used for SEM analysis (Table 1).

### Statistical analyses

Statistical analyses were performed using SPSS version 22.0.0.0 and Mplus version 7.4 [25]. First, descriptive statistics, Pearson and Spearman’s rho correlations were performed to examine the distribution of the data and explore statistical significance of univariate relationships between variables. Variables that were not significantly correlated with fatigue severity and fatigue impact were excluded from SEM analysis.

Second, multivariate analysis of variance (MANOVA) was used to test whether there were differences between visual impairment severity levels and vision-specific, depression and fatigue outcomes. Significant associations were followed up by univariate analyses and pairwise comparisons using the Bonferroni post-hoc test.

Third, a step-wise path model was developed within a SEM framework to investigate whether the differences among visual impairment severity on fatigue could be explained by eye strain and light disturbance, adaptation to vision loss, vision-related mobility and depressive symptoms. In contrast to our previous study [4], most psychosocial and health-related factors were omitted from path analyses because we were primarily interested in vision-related fatigue determinants that could be specific for people with visual impairment. Depression was maintained in the model, as it was associated (medium to high correlations) with all variables of interest. Furthermore, we hypothesized depressive symptoms would mediate the effect of adaptation to vision loss on fatigue [21, 26–28]. Mediation was evaluated based upon the statistical significance of the estimated relative direct, indirect and total effects within each path model. The maximum likelihood estimation based on the delta method was used to calculate direct and indirect effects. This estimation method is robust to non-normality and appropriate for models with continuous and categorical variables [29]. Finally, the significance of relative indirect effects of visual impairment severity were tested with the bias-corrected bootstrapping method proposed by Preacher et al. [30]. A total of 5000 iterations was set to impute 95% bias-corrected confidence interval (BC CI) limits and standard errors for the evaluation of relative indirect effects. In this approach, the indirect effects are deemed significant if the upper and lower bound of the 95% CI does not include 0. Given that visual impairment severity was a multicategorical variable, it was represented in the model by a set of dummy variables created by indicator coding in accordance with the principles of Hayes and Preacher [31]. Severe visual impairment served as the reference category, to which all estimated direct and indirect effects for mild/moderate visual impairment and blindness were compared. These results were therefore described in terms of relative effects. In SEM analyses, a hypothesized model with assumed relationships between fatigue and potential mediating variables was initially tested and further optimized in a step-wise procedure. Model fit was improved by removal of non-significant pathways and by inclusion of additional theoretical pathways based on the modification indices. Each model was assessed using several fit criteria as advised by Wang and Wang [32]: χ^2^-goodness-of-fit, Root Mean Square Error of Approximation (RMSEA < 0.06 represents good fit), the Standardized Root Mean Residual (SRMR < 0.08 represents good fit), the Tucker-Lewis index (TLI > 0.95 represents good fit) and the Comparative Fit Index (CFI > 0.95 represents good fit).

### Sample size calculation

The sample size for this study was determined using commonly accepted rule-of-thumb practices for SEM. Although there is no consensus on exact sample size requirements, previous guidelines have recommended a minimum of 200 participants [33, 34] and a range of 5–20 observations per estimated parameter [35, 36]. Given that our model includes up to 30 estimated parameters, a minimum sample size range of 150 to 600 participants was considered appropriate to ensure robust and reliable results.

## Results

### Participants

Out of 1281 invited patients, 321 agreed to participate and gave written informed consent (response rate 25.1%). Of those, 73 were not eligible (56 were diagnosed/treated for chronic conditions and/or psychiatric disorders, 14 had no visual impairment, 3 were not fluent in Dutch), 10 could not be contacted after multiple attempts and 5 withdrew from participation. In addition, 13 had missing values on essential items for analysis, resulting in data of 220 patients that were included in the present study. The most common reasons reported by non-responders for declining participation were: too much of a burden to participate, not interested and already participating in another study. Specific information on the eye examination dates was missing for nearly half of the study sample, limiting our ability to precisely calculate the time interval between these assessments and study participation. Out of 110 participants with complete data, more than half (71%) had their eye examinations conducted within approximately one year before or after participation in our study.

Table 2 shows the sociodemographic characteristics of the study sample. Retinitis pigmentosa (26.8%) and age-related macular degeneration (24.5%) were the most common causes of visual impairment, followed by glaucoma (13.2%) and homonymous hemianopia (8.2%). The majority of participants (72.7%) reported a progressive disease course with declining visual acuity and/or increasing visual field problems.

**Table 2:** Sample characteristics and MANOVA results for continuous variables by visual impairment severity

Variables, mean (SD) or percentage	Total (*n* = 220)	Mild/moderate VI (*n* = 96)	Severe VI (*n* = 46)	Blindness (*n* = 78)	*F*	*p*	*ηp2*	*Group differences*^b^
*Dependent*								
Fatigue severity^a^	0.34(0.87)	0.23(0.85)	0.62(0.89)	0.30(0.86)	3.19	.043	.029	2 > 1^*^
Fatigue impact^a^	0.47(1.04)	0.37(1.06)	0.78(0.80)	0.40(1.11)	2.71	.069	.024	
Depressive symptoms^a^	0.44(0.78)	0.30(0.78)	0.75(0.75)	0.43(0.77)	5.39	.005	.048	2 > 1^**^
Adaptation to vision loss^a^	-0.01(0.85)	0.09(0.81)	-0.17(0.86)	-0.02(0.90)	1.43	.241	.013	
Vision-related mobility	49.94 (18.95)	55.67 (18.70)	48.43 (17.67)	43.77 (18.08)	9.66	.001	.082	1 > 3^**^
Eye strain & light disturbance^a^	-0.01(1.03)	-0.02(0.92)	0.38(0.90)	-0.19(1.17)	4.51	.012	.040	2 > 3^**^
*Independent*								
Age	57.45 (14.56)	57.39 (15.45)	58.70 (14.93)	56.77 (13.33)	0.22	.801	.002	
Education in years	11.62(2.93)	11.99(2.77)	11.35(2.91)	11.32(3.12)	1.51	.224	.014	
Years since diagnosis	22.57 (17.71)	17.18 (15.81)	24.30 (18.63)	28.26 (17.63)	9.30	.001	.079	3 > 1^**^
Male gender	37.7%	44.8%	30.4%	22.4%				
Stable disease course	27.3%	35.4%	19.6%	34.4%				

*VI* Visual impairment, *M* Mean, *SD* Standard deviation

^*^Statistically significant (*p* < 0.05)

^**^Statistically significant (*p* < 0.01)

^a^Outcomes expressed in thetas

^b^Bonferroni corrected post-hoc tests

### Preliminary analysis

**Fig. 1:**
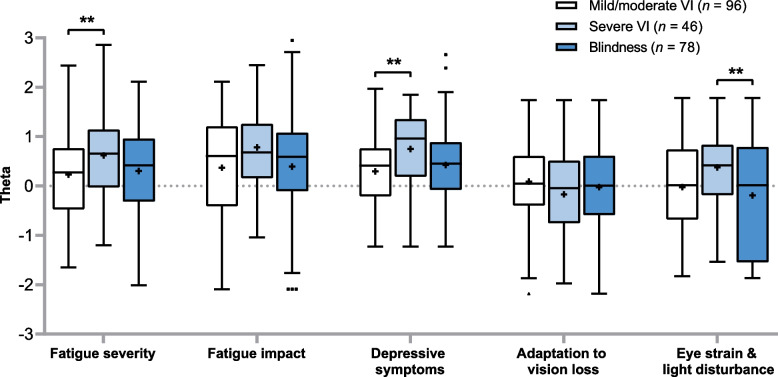
Box plot showing thetas for fatigue, depressive symptoms and vision-specific factors by visual impairment severity category. Boxes display the median and the 25th and 75th percentiles. The plus sign within each box represents the mean. Whiskers and extreme values (dots) were plotted using the Tukey method. Asterisks indicate significant differences between visual impairment severity categories in Bonferroni corrected post-hoc tests. * Statistically significant at *p* < 0.05. ** Statistically significant at *p* < 0.01. *VI* visual impairment

The descriptive statistics confirmed the assumptions of normality and multicollinearity for all study data. As shown in Table 3, there were significant correlations between all potential mediating variables and the dependent fatigue variables. In contrast, none of the independent variables (age, education, gender, years since diagnosis and disease course) were significantly correlated with both fatigue variables, and were therefore excluded from SEM analysis. Sample characteristics and MANOVA results with visual impairment severity as the single factor and all continuous study variables as the dependent variables are presented in Table 2. There was a statistically significant difference in potential mediating variables and dependent fatigue variables based on a patient’s severity of visual impairment, *F* (18, 416) = 3.02, *p* < 0.001; Wilk's Λ = 0.782, partial η^2^ = 0.12. Further analysis with the Bonferroni procedure (statistical significance was accepted at *p* < 0.006) revealed that visual impairment severity had a statistically significant effect on fatigue severity, depressive symptoms, vision-related mobility difficulties and eye strain & light disturbance. Follow-up post-hoc tests indicated that mean fatigue and depressive symptoms were significantly higher for patients with severe visual impairment relative to those with mild/moderate visual impairment, mean levels of eye strain & light disturbance were significantly higher for patients with severe visual impairment compared to those with blindness, and mean vision-related mobility problems were significantly higher for blind patients compared to patients with mild/moderate visual impairment (Fig. 1).

**Table 3:** Correlation matrix of dependent (Y), mediating (X) and independent (E) study variables (*n* = 220)

		Y_1_	Y_2_	X_1_	X_2_	X_3_	X_4_	E_1_	E_2_	E_3_	E_4_	E_5_
Y_1_	Fatigue severity^a^	-										
Y_2_	Fatigue impact^a^	.73^**^	-									
X_1_	Depressive symptoms^a^	.63^**^	.67^**^	-								
X_2_	Adaptation to vision loss^a^	-.35^**^	-.35^**^	-.43^**^	-							
X_3_	Vision-related mobility	-.34^**^	-.32^**^	-.34^**^	.38^**^	-						
X_4_	Eye strain and light disturbance^ab^	.41^**^	.44^**^	.41^**^	-.34^**^	-.35^**^	-					
E_1_	Gender	.10	.18^**^	.11	.06	-.22^**^	.19^§^	-				
E_2_	Age	-.09	.02	-.10	-.20^**^	-.09	.03	.01	-			
E_3_	Education in years	-.09	-.09	-.04	.24^**^	.23^**^	-.23^**^	-.13	-.19^**^	-		
E_4_	Years since diagnosis	.03	-.05	.04	.06	-.14^*^	-.08^**^	.12	-.05	-.01	-	
E_5_	Disease course	-.02	-.02	-.05	-.07	-.14^*^	.25^**^	.01	.23^**^	-.07	-.03	-

Pearson correlations for pairs of continuous variables, Spearman’s rho correlation in case of at least one categorical variable

^*^Statistically significant (*p* < 0.05)

^**^Statistically significant (*p* < 0.01)

^a^Outcomes expressed in thetas

^b^Combined latent variable consisting of two LVQOL items (difficulties with eyes getting tired, difficulties with glare and being dazzled by lights) and one D-Ai item (performing daily activities without suffering from eye discomfort)

### Path analysis

In the initial hypothesized model, three vision-specific potential mediators (eye strain & light disturbance, adaptation to vision loss, vision-specific mobility), one psychosocial potential mediator (depressive symptoms) and two independent dummy variables representing visual impairment severity were included in the model to evaluate their (in)direct relationships with the latent fatigue variable.

As can be seen in Table 4, fit criteria for the initial model were acceptable in terms of CFI and TLI but RSMEA and SRMR exceeded their threshold values of 0.06 and 0.08, respectively (model 1). Because adaptation to vision loss was not significantly related to fatigue and the two dummy variables representing visual impairment severity it was excluded from further analysis. In addition, pathways from D_1_ and D_2_ to depressive symptoms were added to the second model, resulting in good fit across all criteria (Table 4: model 2). In a final effort, removal of insignificant pathways failed to improve fit statistics over the previous model (Table 4: model 3). Hence, model 2 was chosen as our final model, which explained 60% of the latent fatigue variable.

**Table 4:** Model progression and related fit statistics

Model	Fit indices							Steps taken
	${\chi }^{2}$	*df*	RSMEA	90% CI	SRMR	CFI	TLI	
Model 1	86.338	24	0.109	[.084, .134]	0.105	0.903	0.822	Hypothesized model
Model 2	35.061	17	0.069	[.036, .102]	0.071	0.969	0.936	Removal of variable X_2_, inclusion of pathways D_1_ → X_1_ and D_2_ → X_1_
Model 3	53.739	20	0.088	[.060, .116]	0.083	0.942	0.942	Removal of insignificant pathways: D_2_ → X_1_, D_2_ → X_4_, X_3_ → Y

${\chi }^{2}$ Chi square, *df* Degrees of freedom, *RSMEA* Root Mean Square Error of Approximation, *SRMR* Standardised Root Mean Square, *CFI* Comparative Fit Index, *TLI* Tucker-Lewis Index

**Fig. 2:**
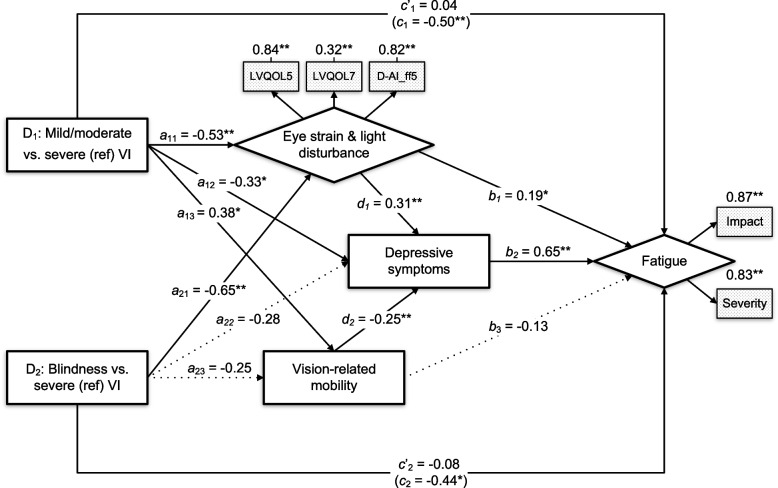
Path analysis output for the final multicategorical SEM model (*n* = 220). Arrows represent direct effects with standardized regression coefficients (StdYX for continuous variables, StdY for categorical variables). Constructs of latent variables (diamond shapes) are shown in dotted boxes. * Statistically significant at *p* < 0.05. ** Statistically significant at *p* < 0.01

A visualization of the final model together with standardized path coefficients of all direct and indirect effects are shown in Fig. 2 and Table 5. Eye strain & light disturbance (pathway *b*_*1*_: β = 0.19, *p* = 0.023) and depressive symptoms (pathway *b*_*2*_: β = 0.65, *p* < 0.001) were directly associated with fatigue. Holding visual impairment severity constant, those who experienced increased symptoms of eye strain & light disturbance and higher levels of depressive symptoms had higher levels of fatigue. Furthermore, eye strain & light disturbance (β = 0.20, 95% BC CI [0.11, 0.30]) and vision-related mobility (β = -0.16, 95% BC CI [-0.25, -0.09]) were significantly associated with the latent fatigue variable through mediation of depressive symptoms. Specifically, higher levels of eye strain and light disturbance, and more problems with vision-related mobility, were associated with higher levels of depressive symptoms (D_1_ and D_*2*_ pathways), which in turn was associated with greater fatigue.

**Table 5:** Standardized path coefficients of the final multicategorical SEM model (*n* = 220)

	Direct effects: β (SE)								Indirect effects: β (SE)		
	Y: Fatigue		X_1_: Depression		X_3:_ Vision-related mobility		X_4:_ Eye strain & light disturbance		*ab*	95% BC CI	
										LL	UL
X_4_: Eye strain and light disturbance	*b*_1_	0.19 (0.08)^*^	*d*_1_	0.31 (0.08)^**^							
Indirect effect: X_4_ → X_1_ → Y									0.20 (0.05)^*^	0.11	0.30
X_3_: Vision-related mobility	*b*_3_	-0.13 (0.07)	*d*_*2*_	-0.25 (0.06)^**^							
Indirect effect: X_3_ → X_1_ → Y									-0.16 (0.04)^*^	-0.25	-0.09
X_1_: Depression	*b*_2_	0.65 (0.05)^**^									
D_1_: Mild/moderate VI vs. severe VI (ref)	*c'*_1_	0.04 (0.13)	*a*_*12*_	-0.33 (0.16)^*^	*a*_*13*_	0.38 (0.18)^*^	*a*_*11*_	-0.53 (0.17)^**^			
Specific: D_1_ → X_1_ → Y									-0.22 (0.11)^*^	-0.44	-0.02
Specific: D_1_ → X_3_ → Y									-0.05 (0.04)^*^	-0.15	-0.01
Specific: D_1_ → X_4_ → Y									-0.10 (0.06)^*^	-0.26	-0.02
Specific: D_1_ → X_3_ → X_1_ → Y									-0.06 (0.04)^*^	-0.15	-0.01
Specific: D_1_ → X_4_ → X_1_ → Y									-0.11 (0.04)^*^	-0.21	-0.04
Relative total indirect effect									-0.54 (0.14)^*^	-0.81	-0.26
Relative total effect	*c*_*1*_	-0.50 (0.18)^**^									
D_2_: Blindness vs. severe VI (ref)	*c'*_2_	-0.08 (0.14)	*a*_*22*_	-0.28 (0.17)	*a*_*23*_	-0.25 (0.17)	*a*_*21*_	-0.65 (0.19)^**^			
Specific: D_2_ → X_1_ → Y									-0.18 (0.11)	-0.41	0.03
Specific: D_2_ → X_3_ → Y									0.03 (0.03)	-0.01	0.13
Specific: D_2_ → X_4_ → Y									-0.13 (0.07)^*^	-0.31	-0.02
Specific: D_2_ → X_3_ → X_1_ → Y									0.04 (0.03)	-0.01	0.11
Specific: D_2_ → X_4_ → X_1_ → Y									-0.13 (0.05)^*^	-0.26	-0.05
Relative total indirect effect									-0.37 (0.17)^*^	-0.69	-0.05
Relative total effect	*c*_*2*_	-0.44 (0.19)^*^									

*β* standardized path coefficient (StdYX for continuous variables, StdY for categorical variables), *SE* standard error, *BC CI* bias corrected confidence intervals (based on 5000 bootstrapped samples), *ref* reference category, *LL* lower limit, *UP* upper limit, *VI* visual impairment

Y: dependent variable, X: mediating variables

D_1_ contrast: mild/moderate visual impairment versus severe visual impairment

D_2_ contrast: blindness versus severe visual impairment

*ab*: product of the direct (*a*) and indirect (*b*) effects on the dependent variable

^*^Statistically significant (*p* < 0.05)

^**^Statistically significant (*p* < 0.01)

Path analysis revealed significant relative total effects of D_1_ and D_2_ on fatigue, indicating that patients with severe visual impairment had significantly higher levels of fatigue compared to those with mild/moderate visual impairment (pathway *c*_*1*_: β = -0.50, 95% BC CI [-0.86, -0.16]) and blindness (pathway *c*_*2*_: β = 0.44, 95% BC CI [0.07, 0.80]). In contrast, the direct effects of D_1_ and D_2_ on fatigue were non-significant when controlling for all other variables in the model. This finding indicates that the visual impairment severity-fatigue association is completely mediated by the other variables included in our model. Bias-corrected bootstrap analysis identified a significant relative total indirect effect of D_1_ (β = -0.54, 95% BC CI [-0.81, -0.26]) and D_2_ (β = -0.37, 95% BC CI [-0.69, -0.05]) on fatigue, accounting for 93% and 83% of their relative total effects, respectively (Table 5).

Eye strain & light disturbance, depressive symptoms and vision-related mobility were all identified as mediators in the link between fatigue and visual impairment severity for the D1 contrast. Relative to those with mild/moderate visual impairment, patients with severe visual impairment had significantly higher levels of depressive symptoms and symptoms of eye strain & light disturbance, and more problems with vision-related mobility, which in turn was associated with increased fatigue symptoms (see Table 5). In addition, the indirect effects of eye strain & light disturbance and vision-related mobility were also sequentially mediated by depression (see Table 5). As for the D2 contrast, the bias-corrected bootstrap analysis showed that the indirect association with fatigue was mediated by eye strain & light disturbance, and by serial mediation of eye strain & light disturbance via depressive symptoms (see Table 5). In other words, compared to patients with blindness, patients with severe visual impairment experienced elevated fatigue levels via higher symptoms of eye strain and light disturbances and related depression.

## Discussion

The present study served two purposes: to test visual impairment severity, eye strain & light disturbances, adaptation to vision loss, vision-related mobility problems as determinants of fatigue, and to examine whether the association between visual impairment severity and fatigue would be mediated by these vision-specific factors and depressive symptoms. To this end, a well-fitting SEM was developed that explained 60% of the variance in fatigue severity and impact on daily life.

With regard to our primary aim, one of the most important findings was the direct association between eye strain & light disturbance and fatigue. Since optimal lighting conditions are essential for improving visual acuity and contrast sensitivity for persons with low vision [37], light disturbances may lead to fatigue as compensatory efforts might be needed to establish visual perception. Besides, it is possible that persons with visual impairment need to invest additional mental resources to counteract focusing problems and accommodative dysfunctions of the eye, potentially leading to excessive strain and fatigue. Similar hypotheses have been formulated to explain the increased levels of fatigue frequently observed in persons with hearing impairments [38]. In these studies, fatigue is often linked to an increased cognitive load, resulting from the extra effort necessary to process degraded speech and auditory signals [39–41]. However, the extent to which mental effort influences fatigue remains disputed and the various mechanisms involved are not fully understood.

Another important finding from our study was the strong influence of depressive symptoms on fatigue. Depression was not only a direct determinant of fatigue with the largest effect size of all variables, it also mediated the indirect associations of vision-related mobility and eye strain & light disturbance with fatigue. These findings are consistent with previous modelling studies in multiple sclerosis [42] and rheumatoid arthritis [43], in which depression has been considered one of the most prominent determinants and mediating factors of fatigue. Psychological interventions that focus on depression, such as cognitive behavioural therapy [44], may therefore also be beneficial in fatigue management of people with visual impairment.

Contrary to expectations, adaptation to vision loss was unrelated to fatigue and was not found to be a significant mediator. However, it did have an indirect effect through depressive symptoms in the first hypothesized model, with better adaptation predicting less depression which in turn decreased fatigue. One possible explanation is that the direct effects of depression and eye strain & light disturbance on fatigue were much greater than that of adaptation to vision loss.

The second aim of the present study involved the association between visual impairment severity, fatigue and potential mediation by vision-specific factors and depression. In general, patients with severe visual impairment reported the highest levels of fatigue severity and fatigue impact on daily life, whereas fatigue levels of patients with blindness were comparable to those with mild/moderate visual impairment. Similar results have been reported by Cypel et al. [12], in which fatigue symptoms were the lowest for older adults with blindness, but seemed to increase with a greater degree of vision loss. Taken together, these findings indicate that the association between fatigue and visual impairment severity follows an inverted U-shape. Several explanations for this complex relationship seem to arise from our modelling as well.

Our results showed that the association between visual impairment severity and fatigue was fully mediated by an interplay of vision-related factors and depression. Eye strain & light disturbance was found to be an important mediator in the relationship between visual impairment severity and fatigue for both contrasts. A possible explanation for the specific impact of eye strain & light disturbance on fatigue in severe visual impairment might be that these patients rely heavily on their residual vision and therefore likely use it as much as possible. Vision-related mobility problems and depressive symptoms on the other hand, only explained variations in fatigue between patients with mild/moderate and severe visual impairment. The observation that mobility problems and depression no longer contribute to fatigue in patients with blindness suggests that some form of adjustment or coping may occur once visual decline stabilizes or cannot deteriorate any further. However, our SEM did not provide evidence for such a mediating role of adaptation to vision loss in the relationship between visual impairment severity and fatigue. All things considered, our findings indicate that the elevated levels of fatigue patients with severe visual impairment are not a direct result of decreased visual acuity and/or increased visual field problems, but could rather be explained through the consequences of these limitations. Supportive evidence for this possibility comes from previous path analysis studies in which the effect of visual impairment on mental health outcomes was largely explained by physical and psychosocial factors [45–47].

The findings of the present study are subject to several limitations. First of all, the cross-sectional design prevents us from inferring a causal order of the associations in our model. Although the assumptions of our model were based on a theoretical framework from previous studies among populations with visual impairment, it is important for future research to test the suggested causal pathways within a longitudinal design. A second limitation of the present study pertains the use of self-report measures only. For future studies, performance based measure such as accelerometers [48] or mobility courses [49] for vision-related mobility and the use of stray light meters as a proxy for disability glare and light disturbances, might provide some more objective insight into actual performance on vision-related measures and fatigue [50]. Fourth, the participation rate of our study was relatively low (25.1%), which may have introduced selection bias. Finally, a notable limitation is the large proportion of incomplete data regarding the time intervals between visual acuity assessments and the administration of questionnaires. This lack of precise timing data may affect the interpretation of the association between visual impairment severity and the study outcomes. However, for the 110 participants with complete data, the majority (71%) underwent their eye examination within a year before or after participating in our study. This timeframe provides some reassurance about the validity of the visual acuity measures in relation to the study outcomes. A strength of the present study is the use of visual field data in addition to visual acuity outcomes which added to the accuracy of visual impairment categorization. Another strength is the application of IRT models to optimize the psychometric properties for the majority of our outcome measures. Furthermore, the statistical advantages of SEM analysis enabled us to construct a proxy for eye strain & light disturbance in the absence of a reliable outcome measure in the scientific literature. Although this latent variable was defined by relatively few numbers of single-item indicators, it had high factor loadings and proved to be an important determinant of fatigue in patients with low vision. Another methodological strength of SEM analysis was the ability to conceptualize fatigue in terms of both severity and impact on daily functioning.

## Conclusions

Findings from our SEM model indicate that eye strain & lighting disturbances is a vision-specific determinant of fatigue in patients with low vision regardless of the degree of visual impairment severity. Depression was also a strong direct determinant of fatigue and fully mediated the indirect effect of vision-related mobility and adaptation to vision loss on fatigue. Furthermore, our study suggests that patients with severe visual impairment may experience increased levels of fatigue compared to patients with mild/moderate visual impairment and blindness due to higher levels of eye strain & light disturbance. In contrast, the influence of vision-related mobility and depression on fatigue seems to vary by level of visual impairment.

The factors identified by our model provide key elements that can be targeted by future studies when developing treatment options for vision-related fatigue. Our findings suggest that multifaceted interventions aimed at improving underlying symptoms, such as depression and light disturbances, may support adults with visual impairment in coping with fatigue. Findings from a recent usability study showed that a newly developed vision-specific eHealth intervention based on behavioural therapy and self-management has the potential to reduce fatigue severity and fatigue impact in patients with low vision [51]. Moreover, including screening instruments of depression, (eye) fatigue and lighting disturbances during the intake and early stages of rehabilitation may identify vulnerable patients at risk of developing severe fatigue.

### Supplementary Information

Supplementary Material 1.

## Data Availability

The data sets used and analyzed in the current study are available from the corresponding author on reasonable request.

## Abbreviations

BC CI: Bias-corrected confidence interval
SEM: Structural equation modelling
WHO: World Health Organization
VA: Visual acuity
MMSE: Mini Mental State Examination
FAS: Fatigue Assessment Scale
MFIS: Modified Fatigue Impact Scale
PHQ-9: Patient Health Questionnaire
AVL-9: Adaptation to Vision Loss questionnaire
IRT: Item response theory
LVQOL: Low Vision Quality of Life questionnaire
D-AI: Dutch ICF Activity Inventory
MANOVA: Multivariate analysis of variance
RMSEA: Root Mean Square Error of Approximation
SRMR: Standardized Root Mean Residual
TLI: Tucker-Lewis Index
CFI: Comparative Fit Index
