# Supplementary material for: The multiple mediating effects of vision-specific factors and depression on the association between visual impairment severity and fatigue: a path analysis study
Source: BMC Psychiatry. 2024 Aug 21;24:572. doi: 10.1186/s12888-024-06014-5 (PMC11337564; doi:10.1186/s12888-024-06014-5)
Supplement: Supplementary file 1 — Supplementary Material 1. [file 12888_2024_6014_MOESM1_ESM.docx]

| **Supplement 1** Characteristics of the finalized outcome measures adjusted by IRT analyses used in this study | | | | | | | | | |
| --- | --- | --- | --- | --- | --- | --- | --- | --- | --- |
|  | **Fit indices** | | | | | |  | **Adjustments**^a^ | |
|  | M2 | RMSEA^b^ | SRMR^c^ | TLI^d^ | CFI^e^ | Theta range |  | Items deleted | Collapsed categories |
|  |  |  |  |  |  |  |  |  |  |
| Fatigue Assessment Scale (FAS) | 0.194 | 0.033 | 0.078 | 0.973 | 0.991 | -2.01, 2.86 |  | 2 | 4 + 5 |
| Modified Fatigue Impact Scale (MFIS) | <0.001 | 0.068 | 0.073 | 0.964 | 0.970 | -2.09, 2.95 |  | 3 | 4 + 5 |
| Patient Health Questionnaire (PHQ-9) | <0.001 | 0.070 | 0.072 | 0.935 | 0.961 | -1.23, 2.66 |  | 1 | 3 + 4 |
| Adaptation to vision loss questionnaire (AVL) | <0.001 | 0.060 | 0.057 | 0.938 | 0.959 | -2.18, 3.92 |  | 0 | 1 + 2 |
| Low Vision Quality of Life Questionnaire (LVQOL)^f^ | - | - | - | - | - | - |  | - | - |
|  |  |  |  |  |  |  |  |  |  |
| ^a^ Items with >40% missing values or >70% scores in lowest or highest response categories or with inter-item correlation >0.7 were deleted. Person theta parameters were computed for each participant which represent an interval score of the underlying latent trait (negative theta’s represent less ability of the latent trait and positive theta’s indicate more ability of the latent trait)  ^b^ *RMSEA* Root Mean Square Error of Approximation (values ≤0.06 represent good fit)  ^c^ *SRMR* Standardized Root Mean Square of Residuals (values ≤0.08 represent good fit)  ^d^ *TLI* Tucker-Lewis Index (values ≥0.95 represent good fit)  ^e^ *CFI* Comparative Fit Index (values ≥0.95 represent good fit)  ^f^ IRT analysis could not be performed for LVQOL due to violation of unidimensionality, local independence and monotonicity assumptions. Instead, summary scores were used in SEM analysis as is commonly done when interval scaling is not an option | | | | | | | | | |
